# Differences in Word‐List Learning Strategies in Amnestic and Non‐Amnestic Mild Cognitive Impairment

**DOI:** 10.1002/alz70857_104490

**Published:** 2025-12-25

**Authors:** Catherine Bosyj, Kristoffer Romero, Ana Badal, Renée K Biss

**Affiliations:** ^1^ University of Windsor, Windsor, ON, Canada; ^2^ York University, North York, ON, Canada

## Abstract

**Background:**

Analyzing strategy‐use during neuropsychological testing may provide novel, sensitive measures of cognitive decline in neurodegenerative disease. On word‐list learning tasks, strategies can include semantic clustering (i.e., recalling semantically‐related items consecutively), or temporal contiguity (i.e., recalling items in the order in which they were presented). While reduced temporal contiguity has been shown to predict cognitive decline, it is unknown whether its use differs across subtypes of mild cognitive impairment (MCI) or how it relates to semantic strategies. We examined recall output on a word‐list learning task by comparing temporal contiguity and semantic clustering across patient groups.

**Method:**

Recall on the Word‐List subtest of the Kaplan‐Baycrest Neurocognitive Assessment was analyzed for 194 patients referred to a memory clinic. Included patients met criteria for amnestic MCI (aMCI; n = 65), non‐amnestic MCI (naMCI; n = 42), or had cognition within normal limits (controls; n = 87). Temporal contiguity scores were calculated as average lag between successively recalled words, adjusting for overall recall and the word's original place in the list. Semantic clustering was calculated as average consecutive recall of categorically related words, controlling for recall performance and chance clustering. Temporal contiguity and semantic clustering were averaged across four learning and one delayed recall trial.

**Result:**

There were significant group differences in temporal contiguity (*p* = 0.02), with naMCI patients less likely to use a temporal contiguity strategy to organize recall relative to control, but not aMCI. Group differences in semantic clustering did not reach significance. Participants using temporal contiguity in recall were less likely to use semantic clustering. In aMCI, use of a temporal contiguity strategy was associated with worse immediate recall, whereas semantic clustering was associated with better immediate and delayed recall. The latter finding also observed in controls.

**Conclusion:**

aMCI participants used temporal contiguity to organize their recall, but this represented a suboptimal strategy relative to semantic clustering. Individuals with aMCI and relatively intact semantic processing may be able to sustain memory performance by adopting semantic strategies. In naMCI, there was less use of temporal contiguity to organize recall, which may be consistent with reduced frontal memory organization abilities.

